# Characterization of workers or population percentage affected by low-back pain (LPB), sciatica and herniated disc due to whole-body vibrations (WBV)

**DOI:** 10.1016/j.heliyon.2024.e31768

**Published:** 2024-05-23

**Authors:** J.F. Sánchez-Pérez, B. Comendador-Jimenez, E. Castro-Rodriguez, M. Cánovas, M. Conesa

**Affiliations:** a Department of Applied Physics and Naval Technology, Universidad Politécnica de Cartagena, Spain; b General Directorate of Pharmacy and Health Products. Conselleria de Sanidad Universal y Salud Pública. Comunitat Valenciana. Spain; c Department of Metallurgical and Mining Engineering, Universidad Católica del Norte, Chile

**Keywords:** Whole-body vibrations (WBV), Daily vibration exposure A(8), Sciatica, Low-back pain, Herniated disc, Dimensionless Probit number

## Abstract

Whole-body vibrations have several harmful effects on the population's health. The most suitable way to characterize the vibrations is to use the daily vibration exposure A (8) and Vibration Dose Value as specified in *Directive 2002/*44/EC. Therefore, based on the existing literature, we propose Probit equations that allow us to relate the population percentage affected by the vibration effects (low-back pain, sciatica, and herniated disc) with the A (8) and the Vibration Dose Value. It is worth noting that there is a good correlation between the experimental data and the expressions obtained, especially for low-back pain and herniated discs. Once the expressions have been validated, we analyze the limit values given in the aforementioned legislation, showing that the percentage of the affected population is significant for them. Therefore, this study also proposes new limits based on their own definitions, which are more in line with the results shown in the bibliography.

## 1 Introduction

According to Convention 148 on the working environment, the term vibrations includes any vibration transmitted to the human body by solid structures that are harmful to health or entail any other type of danger [1]. When a vibration comes into contact with the human body, it transmits energy to it, which is absorbed by the body and can produce certain effects. The organism presents physiological mechanisms capable of perceiving external stimuli that allow it to react to counteract certain harmful effects, such as pupil contraction or the production of melanin against sunlight. In the case of vibrations, the body presents three mechanisms to detect vibrations: the cutaneous, kinesthetic, and vestibular systems [2]. The population can be exposed to vibrations from different sources. The main ones are occupational exposure or exposure through a means of transport: either drivers or passengers traveling by air, land, or sea. Moreover, vibrations can be transmitted in places of residence or leisure [3].

In the early eighteenth century, Ramazzi, founder of occupational medicine, established that there were signs of a relationship between vibration and health [4]. However, until the early 1950s, a relationship between the vibration transmitted by the whole body (WBV) and health problems had not been sought [5]. We had to wait until around 1970 for epidemiological studies to reveal a relationship between occupational factors and musculoskeletal problems [6]. Thus, based on the literature, the main effects on health of vibration exposure depending on the entry route can be classified as transmission to the hand-arm system (HAV) or whole-body (WBV). In this way, *Directive 2002/*44/EC *of the European Parliament and of the Council* defines them as [7].

• *“hand-arm vibration’: the mechanical vibration that, when transmitted to the human hand-arm system, entails risks to the health and safety of workers, in particular vascular, bone or joint, neurological or muscular disorders;”*
• *“whole-body vibration’: the mechanical vibration that, when transmitted to the whole body, entails risks to the health and safety of workers, in particular lower-back morbidity and trauma of the spine.”*

By the one hand, vibrations transmitted to the hand-arm system can present acute effects: activity disturbance or discomfort; and chronic effects: vascular alterations (Raynaud phenomenon or white finger), neurological disorders or musculoskeletal disorders [8]. On the other hand, vibrations transmitted to the whole body (WBV) present as acute effects: discomfort, activity interference, and vision alteration; and as chronic effects, with greater risk to health: low-back pain (LBP), herniated disc, sciatica (nervous system) [9], and less likely alterations in the vestibular system or alterations in reproductive organs and digestive system [10].

Epidemiological literature shows a direct relationship between continuous intensive exposure to whole-body vibrations (WBV) and musculoskeletal disorders. Although the weight that other factors may have in the appearance of such disorders it cannot be ignored, environmental factors such as poor postural position or repetitive weight lifting are highlighted, and physiological factors such as age, smoking, or stress [11,12]. The severity of health problems will largely depend on the dose, which is composed of magnitude and exposure time. In the case of low-back pain (LBP), the exposure time was the main influencing factor; however, in the case of sciatica, the magnitude, without forgetting, of course, it is the combination of these factors as a whole that characterizes this severity [13].

It must be emphasized that vibrations can also provide beneficial effects; in fact, they are frequently used as sports training to reduce back pain [14], as a relaxation system through massages that activate blood circulation, or even for therapeutic purposes (facilitating the expulsion of kidney stones or stimulating intestinal motility) [3].

In this study, to characterize all effects on the population that produce the vibrations, we use the PROBIT methodology (PROBability unIT). This methodology, established by Finney [15], facilitates the population or object percentage affected by an effect or damage through a simple relationship between the dimensionless PROBIT number (Y) and the magnitude of the effect or damage. This methodology has been used to establish the percentage of population or objects affected by a certain effect, such as tympanic rupture or structural damage in buildings caused by overpressures in explosions, burns by solar radiation, and fire [[16], [17], [18], [19], [20], [21], [22]].

Therefore, the aim of this work is to apply the Probit methodology to obtain the percentages of the population affected by low-back pain (LBP), herniated disc, and sciatica of those who have been exposed to vibrations transmitted to the whole body (WBV).

## 2 Methodology

The methodology used will be described below, ranging from the selection of the magnitudes that represent the vibrations transmitted to the whole body (WBV), the search for experimental data that relates the magnitude with the effect, in this case low-back pain (LBP), herniated disc, and sciatica; and finally, the expressions that relate the magnitude with the percentage of the population affected by a given damage.

### 2.1 Frequency and acceleration in vibrations

Mechanical vibration is the periodic movement of particles of a dynamic system around their equilibrium point. From a macroscopic point of view, mechanical vibrations are elastic waves that propagate through a solid system produced by the application of an external force or by the displacement of a system part from its equilibrium point. Consequently, these vibrations can occur in a body subjected to external or internal forces. Therefore, whole-body vibrations (WBV) can appear when they are produced by contact with a surface, such as a vehicle seat or vibrating surfaces.

**Fig. 1:**
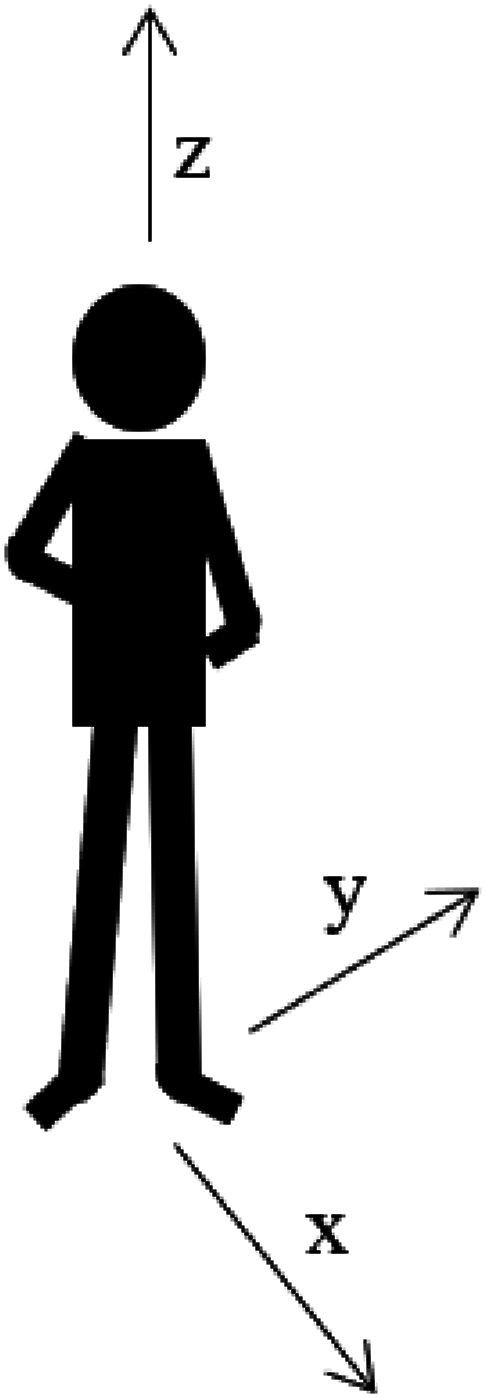
Vibration axis.

Vibration is defined by its magnitude (vibration displacement (m), vibration velocity (m/s), or vibration acceleration (m/s^2^) and frequency (Hz). The magnitude traditionally used to define vibration is acceleration [[23], [24], [25]]. Both the frequency and acceleration can affect health. Thus, for better quantification of its effect, the vibration acceleration and frequency must be measured along the three axes of space (Fig. 1).

The damage produced by vibrations on the body depends on the frequency; therefore, the frequency ranges between 0.1 and 0.5 Hz can cause motion sickness in people due to rocking and pitching movements and from 0.5 to 80 Hz related to health, well-being and perception. To quantify the vibration, frequency-weighted acceleration is developed (equation (1)) [25]:

(1) $$a_{W}=\sqrt{\sum_{i}{\left(W_{i,}a_{i}\right)}^{2}}$$

where a_w_ is the frequency-weighted acceleration in m/s^2^, a_i_ is the acceleration effective value (r.m.s.) for the one-third octave i-th band in m/s^2^, and W_i_ is the weighting factor for the one-third octave i-th band. For the axes x and y, take the value of W_d_ and for the z axis the value of W_k_ given in Table 3 of the *ISO 2631-1 “Mechanical vibration and shock”* [25]. Finally, the total value of the frequency-weighted acceleration of vibration, calculated with the vibration acceleration in each orthogonal coordinate, is given by equation (2):

(2) $$a_{v}=\sqrt{k_{x}^{2}a_{wx}^{2}+k_{y}^{2}a_{wy}^{2}+k_{z}^{2}a_{wz}^{2}}$$

where k is the multiplication factor of each axis. In this way, the multiplying factors are taken to value of 1.4 for axes x and y, unity value for z axis, (x axis: W_d_,k = 1.4, y axis: W_d_,k = 1.4, and z axis: W_k_,k = 1) [[23], [24], [25]].

### 2.2 Daily vibration exposure A (8) and vibration dose value (VDV)

For the health effects evaluation produced for whole-body vibration, the *European Directive 2002/*44/EC allows assessment using two methods [7,23].

• *Daily vibration exposure A(8): “Daily exposure value normalized to an 8-h reference period A(8), expressed as the square root of the sum of the squares (r.m.s.) (total value) of the frequency-weighted acceleration values, determined on the orthogonal axes a*_*hwx*_*, a*_*hwy*_*, a*_*hwz*_*”. The daily vibration exposure A(8) is measured in m/s*^*2*^*.*
• *Vibration Dose Value (VDV): “The highest vibration dose value (VDV) of the frequency-weighted accelerations, determined on three orthogonal axes (1*.*4a*_*wx*_*, 1*.*4a*_*wy*_*, a*_*wz*_*for a seated or standing worker)”. VDV is a cumulative dose and is calculated with the 4*th *root-mean-quad of acceleration signal and its units are m/s*^*1.75*^ [23].

#### 2.2.1 Daily vibration exposure A (8)

The calculation of the daily vibration exposure A (8) must be made for each axis, considering all the vibrations to which people or workers are exposed during the day. Thus, for each axis, it is given by equations (3), (4), (5), (6), (7), (8):

(3) $$A_{x,j}\left(8\right)=1.4a_{wx,j}\sqrt{\frac{T_{exp,j}}{T_{0}}}$$

(4) $$A_{y,j}\left(8\right)=1.4a_{wy,j}\sqrt{\frac{T_{\exp,j}}{T_{0}}}$$

(5) $$A_{z,j}\left(8\right)=a_{wz,j}\sqrt{\frac{T_{\exp,j}}{T_{0}}}$$

(6) $$A_{x}\left(8\right)=\sqrt{\sum_{j}{A_{x,j}\left(8\right)}^{2}}$$

(7) $$A_{y}\left(8\right)=\sqrt{\sum_{j}{A_{y,j}\left(8\right)}^{2}}$$

(8) $$A_{z}\left(8\right)=\sqrt{\sum_{j}{A_{z,j}\left(8\right)}^{2}}$$

where subscript j refers to the different vibration sources to which the person is exposed, T_exp_ is the exposure time to each vibration source, and T_0_ is the reference time of 8 h. Finally, the daily vibration exposure A (8) is the maximum value among those calculated for each axis (equation (9)).

(9) $$A\left(8\right)=\max\left(A_{x}\left(8\right),A_{y}\left(8\right),A_{z}\left(8\right)\right)$$

#### 2.2.2 Vibration dose value (VDV)

To calculate the Vibration Dose Value (VDV), as for A (8), it must be calculated for each axis, considering all the vibrations to which people or workers are exposed. However, for the calculation of the VDV, the time weighting of the exposure time is carried out with the measurement time, unlike for A (8), which uses the time weighting for the 8-h workday [23].The VDV can be calculated with the following expressions (equations (10), (11), (12), (13), (14), (15))):

(10) $${VDV}_{\exp,x,j}=1.4\;{VDV}_{x,j}\sqrt[{4}]{\frac{T_{\exp,j}}{T_{meas,j}}}$$

(11) $${VDV}_{\exp,y,j}=1.4{\;VDV}_{y,j}\sqrt[{4}]{\frac{T_{\exp,j}}{T_{meas,j}}}$$

(12) $${VDV}_{\exp,z,j}={VDV}_{z,j}\sqrt[{4}]{\frac{T_{\exp,j}}{T_{meas,j}}}$$

(13) $${VDV}_{x}=\sqrt[{4}]{\sum_{j}{{VDV}_{\exp,x,j}}^{4}}$$

(14) $${VDV}_{y}=\sqrt[{4}]{\sum_{j}{{VDV}_{\exp,y,j}}^{4}}$$

(15) $${VDV}_{z}=\sqrt[{4}]{\sum_{j}{{VDV}_{\exp,z,j}}^{4}}$$

where subscript j refers to the different vibration sources to which the person is exposed, subscript exp to exposure, T_exp_ is the exposure time to each vibration source, and T_meas_ is the time at which the vibration measurement is performed. Finally, the Vibration Dose Value (VDV) is the maximum value among those calculated for each axis, as shown in equation (16).

(16) $$VDV=\max\left(\;{VDV}_{x},{VDV}_{y},{VDV}_{z}\right)$$

Vibration Dose Value (VDV_axis,j_) is useful when the vibration wave is 'well behaved'. However, under real conditions, there are intermittent vibrations, shocks, and time-varying conditions. Griffin [26] recommended using the estimated Vibration Dose Value (eVDV_axis,j_), which is calculated from the frequency-weighted acceleration (a_w_). The estimated Vibration Dose Value (eVDV_axis,j_) can be calculated using equations (17), (18), (19):

(17) $${eVDV}_{x,j}=1.4\;a_{wx,j}\sqrt[{4}]{T_{meas,j}}$$

(18) $${eVDV}_{y,j}=1.4{\;a}_{wy,j}\sqrt[{4}]{T_{meas,j}}$$

(19) $${eVDV}_{z,j}=1.4\;a_{wz,j}\sqrt[{4}]{T_{meas,j}}$$

where subscript j refers to the different vibration sources to which the person is exposed, and T_meas_ is the time (in seconds) in which the vibration measurement is made. Next, expressions (10) to (16) must be used, but replace the value of VDV_axis,j_ with that of eVDV_axis,j_ for each axis.

### 2.3 Health risks: low-back pain (LBP), sciatica and herniated disc

As we have seen previously, the main chronic effects on health when exposed to whole-body vibrations (WBV) are low-back pain (LBP), sciatica, and herniated disc [27]. In fact, there are authors who affirm that the risk of developing low-back pain (LBP) or sciatica is twice as high in people exposed to whole-body vibrations (WBV) as in those not exposed [28].

Low-back pain (LBP) is a contracture that appears suddenly or gradually, with pain or discomfort of variable intensity. It appears in the lumbar region, above the buttocks. It can be classified into three groups depending on its duration: chronic (7–12 years), acute (<12 weeks), and subacute (between 6 weeks and 3 months) [29].

Sciatica is understood as a symptom resulting from problems caused by the sciatic nerve, which is born in the lower part of the spine, extends towards the toes, and is the longest nerve in the human body. This nerve controls the strength, sensation, and reflexes of legs and feet. The most characteristic symptoms of sciatica, therefore, are pain, numbness, weakness, and tingling of the entire leg up to the toes, which generally affect only one side of the body.

The spine is composed of 26 articulated bones called vertebrae. Among these, there are pads called intervertebral discs. These discs cushion and hold the vertebrae in place. A herniated disc, as its name suggests, is a protrusion of a disc from its usual position due to breakage or displacement. When this occurs, it can press on the nerves or spinal cord and cause injuries.

Musculoskeletal disorders are the most common cause of serious chronic diseases, affecting hundreds of millions of people worldwide. In 2010, the Global Burden of Disease (GBD) evaluated the low-back pain (LBP), positioning it as the first problem related to disability (DALYs), increasing from 58.2 million life years lost due to disability in 1990 to 83 million in 2010 [30]. In addition, in 2016, a study was carried out of the Global Burden of Disease (GBD), regarding injuries and risk factors, where it was established that the low-back pain (LBP) was one of the five leading causes of global disease [31]. Back pain is the second leading cause of sick leave [32]. In fact, research shows that 10 % of patients with this type of disease do not return to work three months after starting sick leave, assuming a very high cost in both health care and indemnification [33].

As musculoskeletal disorders are not perceived as deadly diseases, unlike cancer or HIV, the importance given to them is relative. If the necessary resources were assigned, morbidity could be reduced, and with it, both the incidence and prevalence of this type of sickness. One study presented the following data: 84 % prevalence of low-back pain (LBP) throughout life, 23 % prevalence of chronic low-back pain (LBP) and 11–12 % of the population with disability [34]. Another study showed that the incidence was higher at 30 years, and the prevalence increased with age [35]. It is essential to reduce these rates because life expectancy is increasing (older population), thus increasing expenses in economic terms. In the US, it is estimated that approximately 100 billion dollars a year are spent on actions related to the low-back pain (LBP) [36]. Therefore, obtaining a tool to detect the appearance of these diseases as soon as possible is of great importance in establishing public health prevention and rehabilitation programs [37].

Several studies have found a relationship between low-back pain (LPB) and daily and repeated vibration exposure in the workplace [38]. A study of 133 pilots established that for an acceleration of 0.48 m/s^2^ in a whole-body vibration, 55 % of them regularly experienced low-back pain (LBP) [39]. Another study found that for a similar acceleration (0.43 m/s^2^), 84 % of 242 drivers experienced lifetime low-back pain (LPB) [40]. Similarly, for an acceleration of 1.06 m/s^2^, 81 % of 1155 tractor drivers experienced lifetime low-back pain (LPB) [41]. For an acceleration of 0.72 m/s^2^ in a whole-body vibration, 31 % of 450 tractor drivers and 19 % of non-exposed workers regularly experienced low-back pain (LPB) [42]. Finally, in the case of whole-body vibration with an acceleration of 0.43 m/s^2^, 84 % of 234 bus drivers and 66 % of maintenance workers suffered lifetime low-back pain (LPB) [43]. Table 1 shows a collection of studies carried out over several years that relate general low-back pain (LPB) and exposure to certain accelerations during the workday. In addition, both the professions of the affected workers and that of the control group are included.

**Table 1:** Percentage of population affected with low-back pain (LPB) exposed to different acceleration in their workday.

Reference	A (8) (m/s^2^)	% of worker with LBP	Occupation	% of control group with LBP	Occupation of the control group
[44,45]	0.31	40	Crane operators	–	–
[43,44]	0.43	84	Bus drivers	66	Maintenance workers
[39,44]	0.48	55	Helicopter pilots	11	Non-flying air-force officers
[44,46]	0.55	56	Subway train operators	36	Tower operators
[42,44]	0.72	31	Tractor drivers	19	Non-exposed workers
[40,44]*	0.82	76	Fork-lift truck drivers	30	Non-exposed workers
[41,44]	1.06	81	Tractor drivers	42	Office workers
* The total percentage of workers affected by low-back pain (LPB) is taken					

As shown in Table 1, there is a good correlation between daily vibration exposure A (8) and the percentage of the affected population. That is, in the majority of exposed cases, increasing the daily vibration exposure A (8) increased the percentage of the affected population. It should be noted that a relationship exists when the exposure time to vibration and the daily life of the individual are considered. This may be because the low-back pain already suffered by the population due to poor posture, as can be seen in the control group in Tables 1 and is accentuated by the vibration to which they are exposed.

On the other hand, there are several studies that relate acceleration and sciatica; for example, a study of 492 subway train operators established that 23 % of them suffered sciatica in the last 12 months [46]. Table 2 shows a collection of studies conducted over several years that relate sciatica and exposure to certain accelerations during the workday. In addition, both the profession of the affected workers and that of the control group are included.

**Table 2:** Percentage of population affected with sciatica exposed to different acceleration in their workday.

Reference	A (8) (m/s^2^)	% of worker with sciatica	Occupation	% of Control group with sciatica	Occupation of the control group
[43,44]	0.43	33	Bus drivers	22	Maintenance workers
[39,44]	0.48	12	Helicopter pilots	6	Non-flying air-force officers
[44,46]	0.55	23	Subway train operators	7	Tower operators
[42,44]	0.72	19	Tractor drivers	13	Non-exposed workers
[40,44]	0.82	22	Fork-lift truck drivers	10	Non-exposed workers
[41,44]	1.06	16	Tractor drivers	4	Office workers

**Table 3:** Percentage of population affected with herniated disc exposed to different acceleration in their workday.

Reference	A (8) (m/s^2^)	% of worker with herniated disc	Occupation	% of Control group with herniated disc	Occupation of the control group
[43,44]	0.43	8	Bus drivers	7	Maintenance workers
[39,44]	0.48	5	Helicopter pilots	4	Non-flying air-force officers
[42,44]	0.72	8	Tractor drivers	5	Non-exposed workers
[41,44]	1.06	7	Tractor drivers	2	Office workers

If these data are analyzed, only some of them have a clear relationship with daily vibration exposure A (8). This may be due to the fact that these studies have been completed through questionnaires, and it is possible that the respondent confused sciatica with back pain.

Finally, there are also studies that relate acceleration with the herniated disc, as a study on tractor drivers established that 8 % had a herniated disc [42]. Table 3 shows a collection of studies conducted over several years that relate herniated discs and exposure to certain accelerations during the workday. In addition, both the profession of the affected workers and that of the control group are included.

Analyzing the data presented, in principle, there is no clear correlation; however, if we study the increase in the percentage of the population suffering from disc herniation due to exposure to vibration, that is, the increase compared to the control group, a clear relationship with the magnitude of the study is shown.

### 2.4 Probit equations to determine low-back pain (LBP), sciatica and herniated disc for repeated exposures

The original idea of the Probit function was given by Chester Ittner Bliss in his paper, where he treated the data of a pest killed by a pesticide as the percentage of pests killed. This method was transcribed by D. J. Finney for toxicological applications [15,47,48]. The method consists of the application of statistical correlations to estimate the unfavorable consequences on the population or other elements vulnerable to physical and/or chemical hazardous events. In statistics, the inverse of the distribution or quantile function associated with the standard normal distribution is called a probit function. Thus, the response of a population (or other elements) to a hazardous physical event is distributed according to a lognormal law [15,48,49]. Consequently, Finney proposed a simple methodology for quantifying damage to populations through the Probit function Y, relating it linearly to the logarithm of the magnitude that causes the damage u [15,48,50] (equation (20)):

(20) $$Y=\frac{\ln\left(u\right)-\mu }{\sigma }+5$$

where μ and σ represent the mean and standard deviation of the distribution. Finally, in consequence analysis, the above equation is usually expressed as shown in equation (21):

(21) Y=α+βln(u)

where α and β are constant parameters for each type of damage, to be fitted by experimental data. Finally, the probability value of the population affected by a damage, P, is calculated by the Probit table given by Finney, Table 4, by the equations proposed below or by the following equation [15,48,51] (equation (22)).

(22) $$P=\frac{1}{\sqrt{2\pi }}\int_{-\infty }^{Y-5}e^{-\frac{1}{2}u^{2}}\text{du}$$

**Table 4:** Probit table. Relationship between the percentage of affected people, P, and the value of the Probit number, Y [15,48,50].

	Entry of units of % of affected											
**Entry of tens of % of affected people**	**%**	**0**	**1**	**2**	**3**	**4**	**5**	**6**	**7**	**8**	**9**	PROBIT function value (Y)
	**0**	–	2.67	2.95	3.12	3.25	3.36	3.45	3.52	3.59	3.66	
	**10**	3.72	3.77	3.82	3.87	3.92	3.96	4.01	4.05	4.08	4.12	
	**20**	4.46	4.49	4.23	4.26	4.29	4.33	4.36	4.39	4.42	4.45	
	**30**	4.48	4.50	4.53	4.56	4.59	4.61	4.64	4.67	4.69	4.72	
	**40**	4.75	4.77	4.80	4.82	4.85	4.87	4.90	4.92	4.95	4.97	
	**50**	5.00	5.03	5.05	5.08	5.10	5.13	5.15	5.18	5.20	5.23	
	**60**	5.25	5.28	5.31	5.33	5.36	5.39	5.41	5.44	5.47	5.50	
	**70**	5.52	5.55	5.58	5.61	5.64	5.67	5.71	5.74	5.77	5.81	
	**80**	5.84	5.88	5.92	5.95	5.99	6.04	6.08	6.13	6.18	6.23	
	**90**	6.28	6.34	6.41	6.48	6.55	6.64	6.75	6.88	7.05	7.33	
	**Entry of decimals of % of affected**											
	**%**	**0.0**	**0.1**	**0.2**	**0.3**	**0.4**	**0.5**	**0.6**	**0.7**	**0.8**	**0.9**	
	**99**	7.33	7.37	7.41	7.46	7.51	7.58	7.65	7.75	7.88	8.09	

The dependent variable P has been set as a random variable according to a normal statistical distribution with a mean value of 5 and a standard deviation of 1, which means that a percentage of 50 % corresponds to a Probit value, Y, of 5 [52].

The Probit methodology, established by Finney [15], has been used in numerous fields to establish a relationship between the magnitude and percentage of the population or objects affected by an effect. Thus, it is worth mentioning the use of this methodology in the effects of explosions, fires, solar radiation, etc., as much in people as in objects (buildings) [[16], [17], [18], [19], [20], [21], [22]].

In the case of vibrations, the associated magnitudes are the daily vibration exposure A (8) and the Vibration Dose Value (VDV). Thus, by adjusting the aforementioned experimental data (Table 1, Table 2, Table 3), Probit equations for low-back pain (LBP), sciatica, and herniated disc can be established. It should be noted that for the aforementioned effects to occur, the population must be repeatedly and continuously exposed to vibrations. These can occur, for example, when workers are always exposed to the same vibration in their workplace or when a traveler assiduously uses a train or bus and experiences the vibration during the journey.

#### 2.4.1 Probit equation for low-back pain (LPB)

**Fig. 2:**
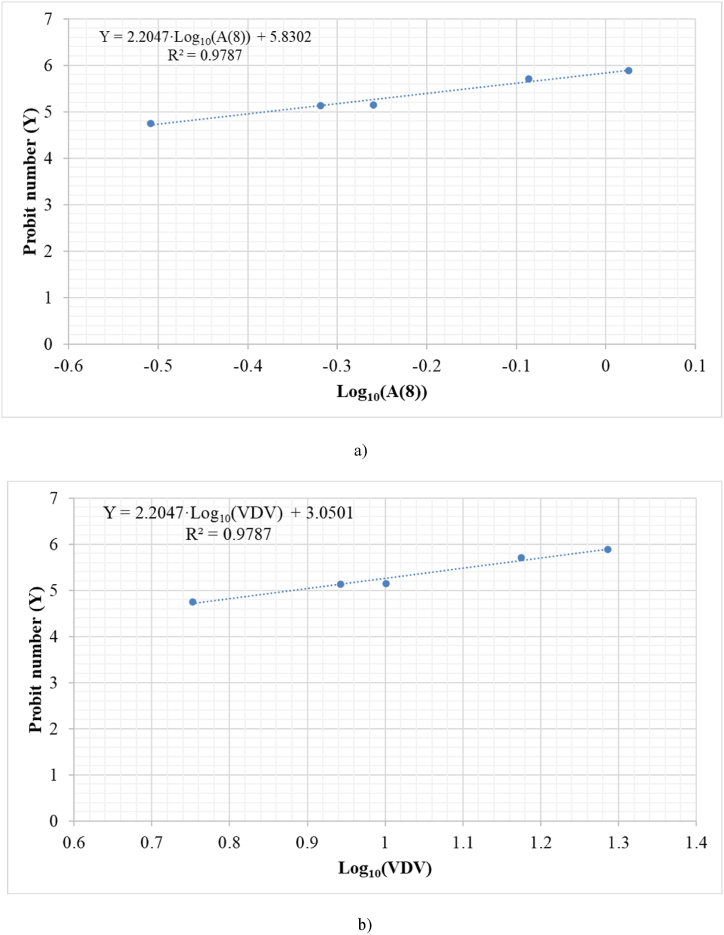
Relationship between the dimensionless Probit number (Y) for low-back pain (LBP) percentages and a) daily vibration exposure A (8) b) Vibration Dose Value (VDV).

Table 1 shows the relationship between low-back pain (LPB) and daily vibration exposure A (8), to which the population is exposed during their working day, and the percentage of the affected population. It should be noted that, as shown in Table 1, a relationship can be seen between the magnitude of daily vibration exposure A (8) and the percentage of the affected population. This percentage includes the effect of the magnitude and workers’ behavior in their daily lives. The affected population percentage can be interpreted by means of the dimensionless Probit (Y) number that assigns a value to each percentage using the Probit table given by Finney, Table 4 [[15], [16], [17], [18], [19], [20], [21], [22]]. Fig. 2 shows the best fit between the dimensionless Probit number (Y) and the daily vibration exposure A (8) using the data in Table 1 and the table relating the value of Y to the percentage of the population affected given by Finney [15]. Analogously, it can be done for the vibration dose value (VDV) using the data in Table 1, the expressions indicated above in Section 2.2.2. and assuming a measurement time of 8 h derived from the information given in the literature.

Equations (23), (24) show Probit equations obtained from the adjustment, both for daily vibration exposure A (8) and for the Vibration Dose Value (VDV). It should be noted that the good adjustment of these expressions provides a good capacity to predict the study magnitude and the percentage of the affected population.

(23) $$Probit\;equation\;for\;low-back\;pain\;as\;function\;of\;A{\left(8\right)\;Y=5.8302+2.2047\;\log}_{10}\left(A\left(8\right)\right)$$

(24) $$Probit\;equation\;for\;low-back\;pain\;as\;function{\;of\;VDV\;Y=3.0501+2.2047\;\log}_{10}\left(VDV\right)$$

#### 2.4.2 Probit equation for sciatica

**Fig. 3:**
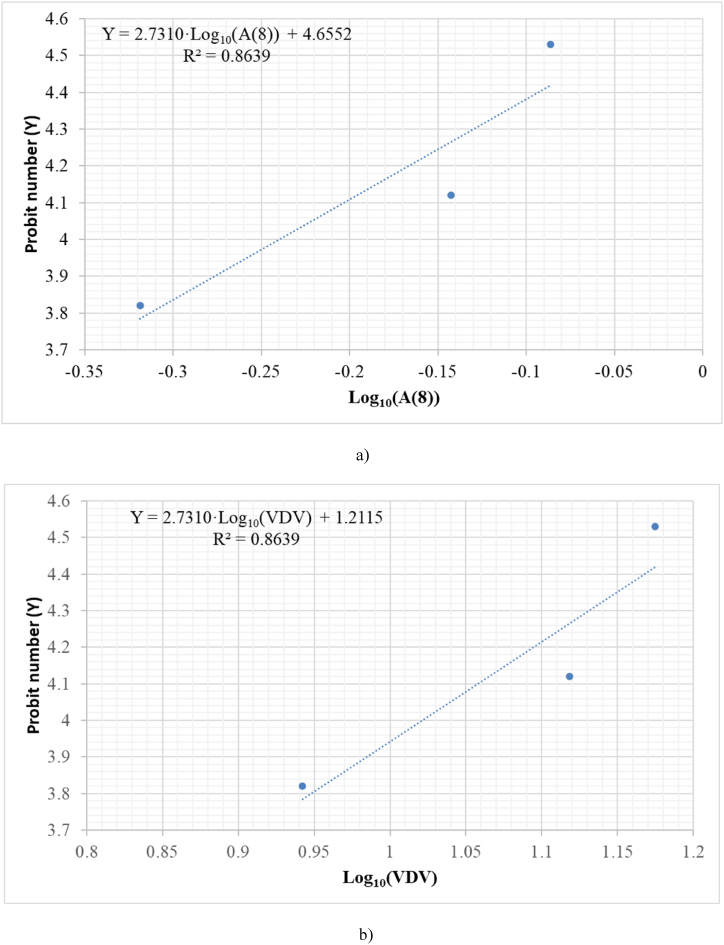
Relationship between the dimensionless Probit number (Y) for sciatica percentages and a) daily vibration exposure A (8) b) Vibration Dose Value (VDV).

Table 2 shows the relationship between the population percentage affected by sciatica, the study magnitude, and the vibration exposure A (8). If the data are analyzed, it can be observed that there is no clear relationship between this magnitude and the percentage of the affected population. An explanation for this may be that these percentages have been created by means of a survey of the study population, and sciatica could have been confused with back pain [54]. Fig. 3 shows the best fit between vibration exposure A (8) and dimensionless Probit number (Y). In the same way, it can be done for the Vibration Dose Value (VDV) using the data in Table 2, the expressions indicated above in Section 2.2.2. and assumed a measurement time of 8 h. Because of this, the adjustment will be made with only three points; therefore, in this case, the equation obtained has this weakness. However, in this study we want to show the application of the Probit methodology, so this adjustment will be used.

Equations (25), (26) show Probit equations obtained from the adjustment, both for daily vibration exposure A (8) and for the Vibration Dose Value (VDV).

(25) $$Probit\;equation\;for\;sciatica\;as\;function{\;of\;A\left(8\right)\;Y=4.6552+2.7310\;\log}_{10}\left(A\left(8\right)\right)$$

(26) $$Probit\;equation\;for\;sciatica\;as\;function\;of\;V{DV\;Y=1.2115+2.7310\;\log}_{10}\left(VDV\right)$$

#### 2.4.3 Probit equation for herniated disc

**Fig. 4:**
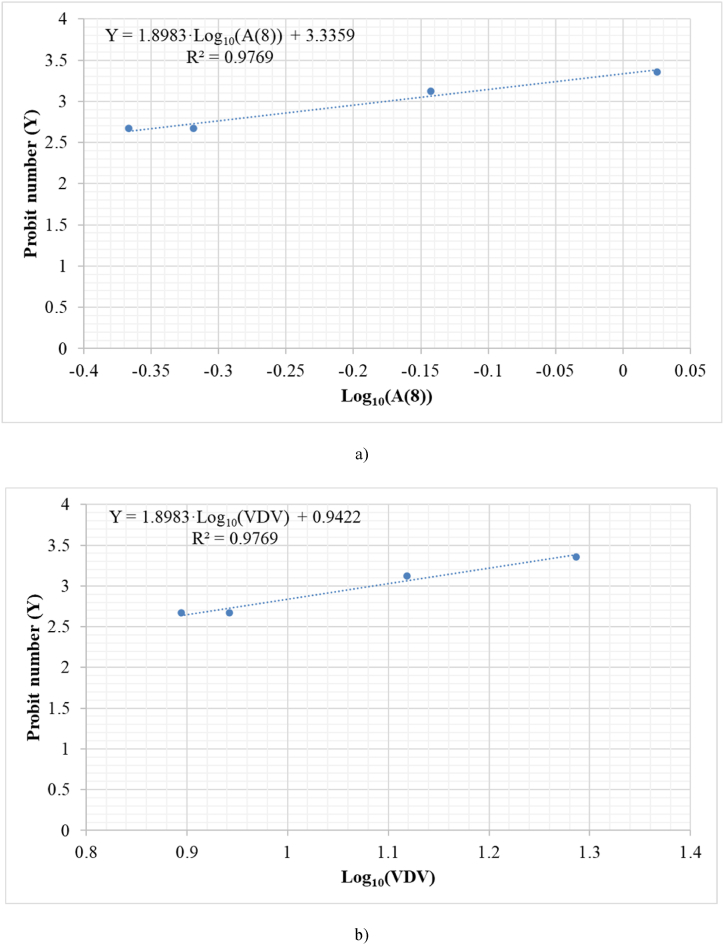
Relationship between the dimensionless Probit number (Y) for percentage increase in suffering from a herniated disc and a) daily vibration exposure A (8) b) Vibration Dose Value (VDV).

Analyzing the data shown in Table 3, we can observe a clear relationship between the percentage increase in the population that would suffer the herniated disc and the magnitude that causes the effect, the daily vibration exposure A (8). Fig. 4 shows the best fit based on the experimental data between the dimensionless Probit (Y) number and daily vibration exposure A (8).

Equations (27), (28) show the Probit equations obtained from the adjustment, both for daily vibration exposure A (8) and for the Vibration Daily Value (VDV). To calculate the VDV, the expressions given in Section 2.2.2 (Table 3), and a time of 8 h were used. It should be noted that these expressions provide the percentage increase in suffering from a herniated disc if the population is exposed to the study magnitudes.

(27) $$Probit\;equation\;for\;herniated\;disc\;as\;function{\;of\;A\left(8\right)\;Y=3.3359+1.8983\;\log}_{10}\left(A\left(8\right)\right)$$

(28) $$Probit\;equation\;for\;herniated\;disc\;as\;function\;of\;V{DV\;Y=0.9422+1.8983\;\log}_{10}\left(VDV\right)$$

#### 2.4.4 Relationship between dimensionless Probit number (Y) and percentage of affected population

The Probit value,Y, obtained from the above expressions can be interpreted through the Probit table (Table 4) [15,48,53], which will provide us with the percentage of the population affected by low-back pain (LBP), sciatica, or herniated disc after repeatedly suffering the vibration that produces the effect. Another way to obtain the percentages of the affected population is by using the following expressions (equations (29)–(31)) obtained from the adjustment of the Probit table (Table 4), which relates the Probit value, Y, to the percentage of the affected population, P, for different ranges of Y:

(29) $$2.67\leq Y<3.59\;P={4.6439Y}^{4}-{54.598Y}^{3}+{245.52Y}^{2}-495.03Y+375.64$$

(30) $$3.59\leq Y<6.9\;P=-{0,096Y}^{6}+{3.5618Y}^{5}-{52.775Y}^{4}+{398.61Y}^{3}-{1615.5Y}^{2}+3355.9Y-2814.1$$

(31) $$6.9\leq Y\leq 8.09\;P=-{0.832Y}^{4}+{26.502Y}^{3}-317.57^{2}+1697.2Y-3313.7$$

where P is the percentage of the population affected.

### 2.5 Expressions for determine percentage of affected population of low-back pain, sciatica and herniated disc for repeated exposures

**Fig. 5:**
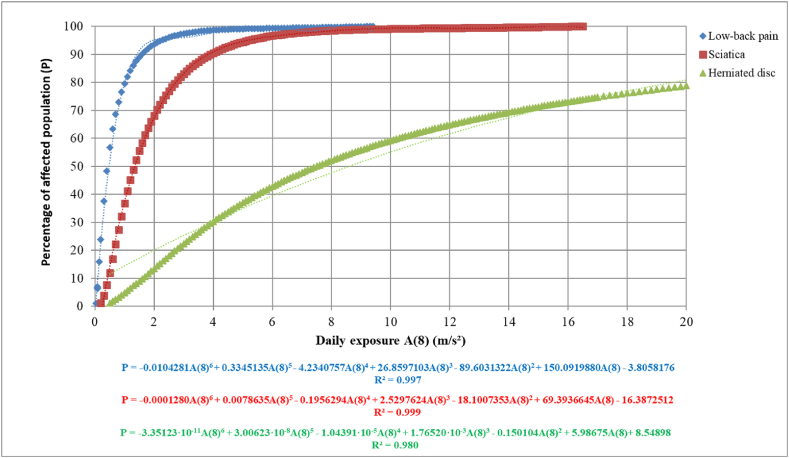
Relationship between the percentage of affected population for low-back pain (LPB), sciatica or herniated disc and daily vibration exposure A (8).

As all expressions described above are connected, an expression that relates the affected population to the magnitude that causes the effect can be obtained. In this way, Fig. 5 represents the daily vibration exposure A (8) against the percentage of the population affected by low-back pain (LBP), sciatica, and herniated disc for repeated exposures using expressions (23), (25), (27), and (29) to (31).

Thus, the expressions that relate the percentage of population affected by low-back pain, sciatica or herniated disc and daily vibration exposure A (8) are obtained (equations (32)–(34)):

Low-back pain (LBP):

(32) $$P=-0.0104281A{\left(8\right)}^{6}+0.3345135A{\left(8\right)}^{5}\;-\;4.2340757A{\left(8\right)}^{4}+26.8597103A{\left(8\right)}^{3}\;-\;89.6031322A{\left(8\right)}^{2}+150.0919880A\left(8\right)\;-\;3.8058176$$

Sciatica:

(33) $$P=-0.0001280A{\left(8\right)}^{6}+0.0078635A{\left(8\right)}^{5}\;-\;0.1956294A{\left(8\right)}^{4}+2.5297624A{\left(8\right)}^{3}\;-\;18.1007353A{\left(8\right)}^{2}+69.3936645A\left(8\right)\;-\;16.3872512$$

Herniated disc:

(34) $$P=-3.35123\cdot 10-11A{\left(8\right)}^{6}+3.00623\cdot 10-8A{\left(8\right)}^{5}\;-\;1.04391\cdot 10-5A{\left(8\right)}^{4}+1.76520\cdot 10-3A{\left(8\right)}^{3}\;-\;0.150104A{\left(8\right)}^{2}+5.98675A\left(8\right)+8.54898$$

**Fig. 6:**
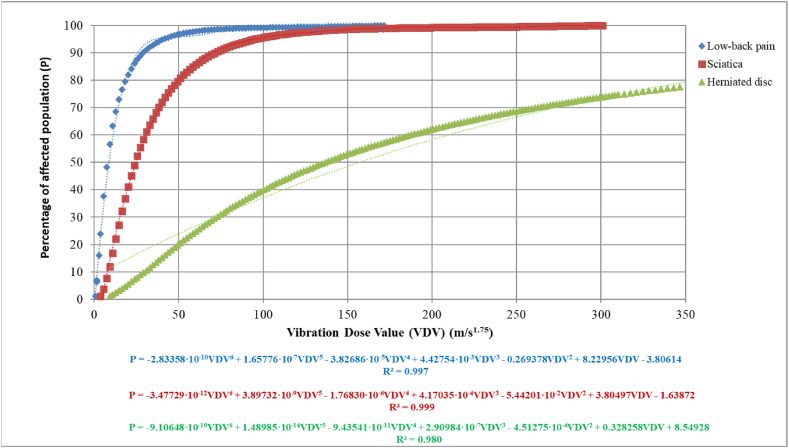
Relationship between the percentage of affected population for low-back pain (LPB), sciatica or herniated disc and Vibration Dose Value (VDV).

Analogously, Fig. 6 represents the Vibration Dose Value (VDV) against the percentage of population affected by low-back pain (LBP), sciatica and herniated disc for repeated exposures using expressions (24), (26), (28) and (29) to (31).

Again, the expressions that relate the percentage of population affected by low-back pain, sciatica or herniated disc and Vibration Dose Value (VDV), assuming a measurement time of 8 h and using the expressions given above in section 2.2.2., are obtained (equations (35)–(37)):

Low-back pain (LBP):

(35) $$P=-2.83358\cdot 10-{10VDV}^{6}+1.65776\cdot 10-{7VDV}^{5}\;-\;3.82686\cdot 10-{5VDV}^{4}+4.42754\cdot 10-{3VDV}^{3}\;-\;{0.269378VDV}^{2}+8.22956VDV\;-\;3.80614$$

Sciatica:

(36) $$P=-3.47729\cdot 10-{12VDV}^{6}+3.89732\cdot 10-{9VDV}^{5}\;-\;1.76830\cdot 10-{6VDV}^{4}+4.17035\cdot 10-{4VDV}^{3}\;-\;5.44201\cdot 10-{2VDV}^{2}+3.80497VDV\;-\;1.63872$$

Herniated disc:

(37) $$P=-9.10648\cdot 10-{19VDV}^{6}+1.48985\cdot 10-{14VDV}^{5}\;-\;9.43541\cdot 10-{11VDV}^{4}+2.90984\cdot 10-{7VDV}^{3}\;-\;4.51275\cdot 10-{4VDV}^{2}+0.328258VDV+8.54928$$

Although all the expressions have a good fit, an R^2^ very close to 1, owing to the sensitivity of these equations to the number of decimal places, it is advisable to use expressions (23) to (28) and subsequently the Probit table given by Finney, Table 4 [15,48], or expressions (29) to (31) to obtain greater precision in the results. This sensitivity is especially significant in the case of obtaining the incremental percentage of people affected by disc herniation, as it is the one with the worst fit (R^2^ = 0.980).

If we study Fig. 5, Fig. 6, it can be observed that to reach a 50 % of the population affected by low-back pain (LBP) or sciatica, a relatively low daily vibration exposure A (8) is needed, around 0.45 m/s^2^ and 1.5 m/s^2^, respectively. However, to reach this value for the incremental percentage of the population affected by herniated disc, a value of approximately 7.5 m/s^2^ would be needed. Comparing the trends of the population affected by low-back pain (LBP) and sciatica, it can be observed that they present a relationship around three times, that is, to reach the same percentage for each of these effects; it takes approximately three times more daily vibration exposure A (8) for sciatica than for the low-back pain (LBP). If we study the trend for each of the effects caused by vibration, we can see that low-back pain (LBP) has a steeper slope, quickly reaching 90 % of the affected population with a value of approximately 1.6 m/s^2^. In the case of sciatica, the slope is steeper, requiring a value of approximately 4 m/s^2^ to reach 90 % of the affected population. Finally, for the incremental percentage of population affected with herniated disc, as expected, due to the nature of the damage and the data obtained from the literature, it has a less pronounced slope, requiring very high values of A (8) to reach 90 % of the population affected, needing approximately 35 m/s^2^.

However, it should be noted that the expressions presented in this paper are not free from errors, arising both from the adjustments themselves and from the data used.

## 3 Results and study cases: Comparison with the regulations

The *European Directive 2002/*44/EC [7] sets two limits for vibrations: the daily exposure action value and the daily exposure limit value, both standardized to an 8-h reference A (8) or at the choice of the EC Member State, Vibration Dose Value (VDV). The daily exposure action value establishes a value above which the worker's exposure should be controlled, and the daily exposure limit value establishes a value above which the worker should not be exposed [7,23]. For whole-body vibration (WBV), the Directive set a daily exposure action value of 0.5 m/s^2^ for daily vibration exposure A (8) or 9.1 m/s^1.75^ for Vibration Dose Value (VDV). Analogously, it sets a daily exposure limit value of 1.15 m/s^2^ for daily vibration exposure A (8) or 21 m/s^1.75^ for the Vibration Dose Value (VDV).

If we introduce the values of the daily exposure action value and daily exposure limit value into the Probit equations, we obtain the results shown in Table 5.

**Table 5:** Percentage of population affected with low-back pain (LBP), sciatica and herniated disc using daily exposure action value and daily exposure limit value.

	A (8) (m/s^2^)	Percentage of population affected with low-back pain (LPB)	Percentage of population affected with sciatica	Incremental percentage of population affected with herniated disc
Daily exposure action value	0.5	57	12	1
Daily exposure limit value	1.15	83	43	6

That is, based on the expressions obtained, which are not free of error due to the adjustments themselves and the inherent error of each of the studies used to make the adjustment, if a worker is exposed to the daily exposure action value for a long period, 57 % of the population is affected by low-back pain, 12 % by sciatica, and 1 % by an incremental percentage of herniated disc. If they are exposed to the daily exposure limit value, 83 % would suffer low-back pain, 43 % with sciatica, and 6 % of incremental percentage of affected with herniated disc. It should be noted that if we compare the values for low-back pain, the daily exposure limit value affects approximately 1.5 times the population than the daily exposure action value. In the case of sciatica is near to 3.6 times and that for herniated disc is six times. It should be noted that in the case of the daily exposure limit, more than 80 % of the population is affected by low-back pain. It is worth noting that these results are in agreement with those experimentally reported in the literature (Table 1, Table 2, Table 3). However, it should be noted, based on the above tables, that the percentages of the affected population could be lower because, in some cases, the control groups had high percentages of damage.

In our opinion, applying this methodology, the daily exposure action value should be placed at 1 % of the population affected by low-back pain, since it is when the effects begin to appear in the workers and, adhering to their own definition, is when measures must be taken. The daily exposure limit value, on the other hand, should be between 10 % and 50 % of the affected population. It should be emphasized that the expressions presented in this paper were obtained from data compiled from the literature and therefore have an associated error, both in the adjustments themselves and in the error associated with each of the studies on which they are based. Table 6 shows the possible values of daily vibration exposure A (8) or the Vibration Dose Value (VDV) for these percentages.

**Table 6:** Values of daily vibration exposure A (8) or Vibration Dose Value (VDV) for different percentages of population affected by low-back pain (LPB).

Percentage of population affected with low-back pain	A (8) (m/s^2^)	VDV (m/s^1.75^)
1 %	0.0369	0.672
10 %	0.110	2.01
25 %	0.209	3.81
50 %	0.420	7.66
75 %	0.846	15.4
99.9 %	10.6	193

On the other hand, Table 7, Table 8 show values of the daily vibration exposure A (8) or Vibration Dose Value (VDV) for different percentages of population affected by sciatica and incremental percentages of population affected by herniated disc, respectively.

**Table 7:** Values of daily vibration exposure A (8) or Vibration Dose Value (VDV) for different percentages of population affected by sciatica.

Percentage of population affected with sciatica	A (8) (m/s^2^)	VDV (m/s^1.75^)
1 %	0.188	3.42
10 %	0.455	8.29
25 %	0.7605	13.9
50 %	1.34	24.4
75 %	2.35	42.9
99.9 %	18.1	330

**Table 8:** Values of daily vibration exposure A (8) or Vibration Dose Value (VDV) for different incremental percentages of population affected by herniated disc.

Incremental percentage of population affected with herniated disc	A (8) (m/s^2^)	VDV (m/s^1.75^)
1 %	0.446	8.13
10 %	1.59	29.1
25 %	3.34	60.9
50 %	7.53	137
75 %	17.0	309
99.9 %	319	5826

## 4 Conclusions

In this work, we have presented several expressions that allow us to relate the representative variables of whole-body vibrations (WBV) and the percentage of the population affected by their different effects, such as low-back pain (LBP), sciatica, and herniated disc. According to *Directive 2002/*44/EC [7], the magnitudes that best quantify the vibrations, and therefore the most used, are the daily vibration exposure A (8) and Vibration Dose Value (VDV); therefore, in this study, the final expressions are given in terms of these variables.

Based on the experimental data, the Probit expressions that allow us to predict the percentage of the population affected by low-back pain (LBP), sciatica or herniated disc as a function of daily vibration exposure A (8) or Vibration Dose Value (VDV) were adjusted. It should be noted that for both low-back pain (LBP) and herniated disc, there is a good correlation between the experimental data and the expression obtained; however, there is no such good adjustment in the case of sciatica, possibly because in the surveys conducted, the respondents confused it with back pain [54].

Once the proposed expressions were validated, we compared the values established in the regulations. For whole-body vibration (WBV), the *Directive 2002/*44/EC [7] set a daily exposure action value and daily exposure limit value of 0.5 m/s^2^ and 1.15 m/s^2^, respectively, for daily vibration exposure A (8). The first establishes when the worker's exposure must be controlled, and the second establishes when it should not be exceeded. The first limit provides 57 % of low-back pain, 12 % of sciatica, and a 1 % of increased risk of herniated disc, and the second limit was 83 % for low-back pain, 43 % for sciatica, and 6 % for increased risk of herniated disc.

Therefore, based on the definition of these limits, the daily exposure action value should be placed at 1 % of the population affected by low-back pain (LBP), since it is when the effects begin to appear in the workers, and consequently, when measures must be taken. Applying the methodology, 1 % of workers affected by low-back pain corresponded to a value of 0.0369 m/s^2^ for daily vibration exposure A (8) and 0.672 m/s^1.75^ Vibration Dose Value (VDV). That is, it is approximately ten times lower than that marked by the regulation. The daily exposure limit value, on the other hand, we believe that should be between 10 % and 50 % of the affected population, corresponding it a value between 0.110 m/s^2^ and 0.420 m/s^2^ for daily vibration exposure A (8) and a value between 2.01 m/s^1.75^ and 7.66 m/s^1.75^ Vibration Dose Value (VDV).

In conclusion, it is worth highlighting the limitations of this study. On one hand, the expressions presented are not free of error, derived both from the adjustments themselves and from the data used, obtained from the bibliography, where each study would have its own associated error. On the other hand, in the case of sciatica, as there was no clear relationship between A (8) and the percentage of the population affected, the adjustment had to be made with only three points. For the rest of the injuries, the adjustment was made with more points, although it would be advisable, if possible, for future work to include more points for adjustment. In terms of the strengths of this work, it should be noted that a methodology is proposed that allows the percentage of the population affected by vibrations to be calculated for each lesion. In addition, the results obtained were compared with current legislation. Finally, it should be noted that this methodology is currently being used in subjects as diverse as explosion damage or the effect of fungicides on fish [[55], [56], [57], [58]].

### 4.1 Ethical Approval and Consent to participate

Not applicable.

### 4.2 Consent for publication

Not applicable.

### 4.3 Availability of supporting data

The dataset used and analyzed are available from the authors.

### Funding

Not applicable.

## CRediT authorship contribution statement

**J.F. Sánchez-Pérez:** Writing – review & editing, Writing – original draft, Validation, Methodology, Investigation, Formal analysis, Conceptualization. **B. Comendador-Jimenez:** Writing – review & editing, Writing – original draft, Validation, Methodology, Conceptualization. **E. Castro-Rodriguez:** Writing – review & editing, Writing – original draft, Validation, Methodology, Investigation, Conceptualization. **M. Conesa:** Writing – review & editing, Writing – original draft, Validation, Methodology, Investigation, Writing – review & editing, Writing – original draft, Validation, Methodology, Investigation.

## Declaration of competing interest

The authors declare that they have no known competing financial interests or personal relationships that could have appeared to influence the work reported in this paper.
